# Cranial Remain from Tunisia Provides New Clues for the Origin and Evolution of Sirenia (Mammalia, Afrotheria) in Africa

**DOI:** 10.1371/journal.pone.0054307

**Published:** 2013-01-16

**Authors:** Julien Benoit, Sylvain Adnet, Essid El Mabrouk, Hayet Khayati, Mustapha Ben Haj Ali, Laurent Marivaux, Gilles Merzeraud, Samuel Merigeaud, Monique Vianey-Liaud, Rodolphe Tabuce

**Affiliations:** 1 Institut des Sciences de l’Evolution, Université Montpellier 2, Montpellier, France; 2 Office National des Mines, Tunis, Tunisia; 3 Géosciences Montpellier, Université de Montpellier 2, Montpellier, France; 4 Service d'Imagerie Médicale de l’Hôpital Lapeyronie, CHU de Montpellier, Montpellier, France; Texas A&M University-Corpus Christi, United States of America

## Abstract

Sea cows (manatees, dugongs) are the only living marine mammals to feed solely on aquatic plants. Unlike whales or dolphins (Cetacea), the earliest evolutionary history of sirenians is poorly documented, and limited to a few fossils including skulls and skeletons of two genera composing the stem family of Prorastomidae (*Prorastomus* and *Pezosiren*). Surprisingly, these fossils come from the Eocene of Jamaica, while stem Hyracoidea and Proboscidea - the putative sister-groups to Sirenia - are recorded in Africa as early as the Late Paleocene. So far, the historical biogeography of early Sirenia has remained obscure given this paradox between phylogeny and fossil record. Here we use X-ray microtomography to investigate a newly discovered sirenian petrosal from the Eocene of Tunisia. This fossil represents the oldest occurrence of sirenians in Africa. The morphology of this petrosal is more primitive than the Jamaican prorastomids’ one, which emphasizes the basal position of this new African taxon within the Sirenia clade. This discovery testifies to the great antiquity of Sirenia in Africa, and therefore supports their African origin. While isotopic analyses previously suggested sirenians had adapted directly to the marine environment, new paleoenvironmental evidence suggests that basal-most sea cows were likely restricted to fresh waters.

## Introduction

**Figure 1:**
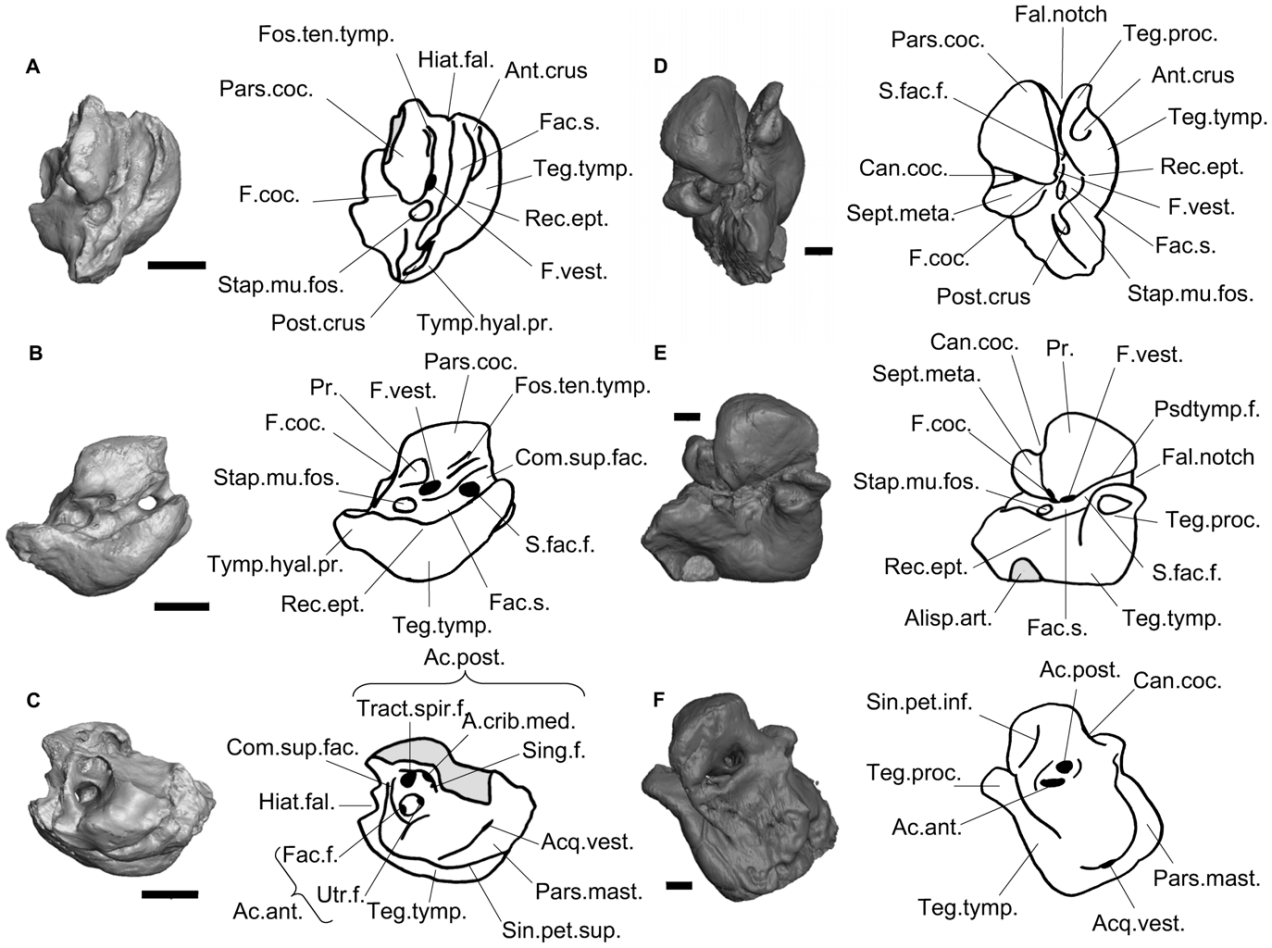
CT reconstruction of the petrosals of the sirenian from Chambi and *Prorastomus*. ABC: CBI-1-542. DEF: *Prorastomus* (BMNH 44897). AD: ventral view, BC: ventrolateral view, CF: dorsal view. Scale bar = 5mm. Broken areas are in grey on drawings. *Prorastomus* was mirrored for the purpose of comparison. Ac.ant.: *foramen acusticum anterius*; A.crib.med.: *area cribrosa media*; Ac.post.: *foramen acusticum posterius*; Acq.vest.: *acqueductus vestibuli*; Alisph.art.: alisphenoid articulation facet; Ant.crus: attachment area for the anterior crus of the tympanic; Can.coc.: *canaliculus cochleae*; Com.sup.fac.: *commissura suprafacialis;* Fac.f.: facial foramen; Fac.s.: facial sulcus; Fal.notch: fallopian notch; F.coc.: *fenestra cochleae*; Fos.ten.tymp.: *fossa tensor tympani*; F.vest.: *fenestra vestibuli*; Hiat.fal.: *hiatus fallopi*; Pars.coc.: *pars cochlearis*; Pars.mast.: *pars mastoidea*; Post.crus: attachment area for the posterior crus of the tympanic; Psdtymp.f.: pseudotympanic foramen; Rec.ept.: *recessus epitympanicus*; Sept.meta.: *Septum metacochleae*; S.fac.f.: secondary facial foramen; Sing.f.: singular foramen; Sin.pet.inf.: *sinus petrosus inferius*; Sin.pet.sup.: *sinus petrosus superius*; Stap.mu.fos.: stapedial muscle fossa; Teg.tymp.: *tegmen tympani*; Teg.proc. : tegmen process; Tract.spir.f.: *tractus spiralis foraminosus*; Tymp.hyal.pr.: tympanohyal process; Utr.f.: utricular foramen.

Sirenia include the living manatees and dugongs, but the widespread and rich fossil record of this order testify to its outstanding past successfulness [1]. Sirenia are deeply nested within Paenungulata, a clade clustering Hyracoidea (dassies), Proboscidea (elephants) and their extinct relatives (e.g., Embrithopoda, Desmostylia) together [2]–[5]. Many of the earliest fossil records of these orders are found in the Paleogene of Africa [2]–[5], which implies that the Paenungulata, including Sirenia, probably shared a common ancestry on the African continent [6]. Molecular phylogenies also support an African root for Paenungulata, considering that they belong to a clade of extant African mammals, the so-called supercohort Afrotheria [2], [5]. Still, while molecular studies support an African origin for sea cows, stem sirenians before the middle Eocene of Africa had so far remained undocumented [5], [6]. In fact, the oldest and most primitive sirenians were exclusively found in the late Early to Early Middle Eocene of Jamaica [7], [8]. These primitive species belong to the family of Prorastomidae, a paraphyletic group consistently appearing as the basal-most offshoot within the Sirenia clade [4], [9]–[11]. Among those “prorastomids”, *Prorastomus* is primarily documented by an almost complete skull from the late Ypresian or early Lutetian (∼48–50 Ma) of Jamaica [7] and, so far, this taxon represented the most ancient and primitive sirenian known. Another genus, from the early Lutetian (∼47–49 Ma) of Jamaica and appearing morphologically advanced over *Prorastomus* [4], [8], [9], *Pezosiren*, is also known by nearly complete skeletal remains but its skull remains undescribed [2]. At last, one putative “prorastomid” was recently found in Senegal [12], but given its young age (late Middle Eocene) and its advanced morphology, this species would not represent a sirenian more primitive than *Prorastomus* [12]. In the end, to date, *Prorastomus* represented the oldest and basal-most sirenian known, and in the absence of more primitive sea cows in Africa, their place of origin remained a true biogeographical and phylogenetic paradox [1], [13]. Here, we describe a new fossil of sirenian, which consists of a petrosal (CBI-1-542) (Fig. 1A, B, C). This fossil was recently found in the late Ypresian – early Lutetian fossil mammal-bearing locality of Djebel Chambi in Tunisia [14], [15]. Interestingly, this African locality is roughly contemporaneous with the Jamaican locality that yielded *Prorastomus*. CBI-1-542 is a left petrosal, half the size of the one of *Prorastomus* (Fig. 1D, E, F, table 1), and represents the earliest cranial remain of a sirenian found in Africa.

**Table 1:** Measurements of the petrosals and bony labyrinth of various Paenungulata. Measurements of *Trichechus* after 21. Linear measurements are in millimeters.

		Petrosal								Bony labyrinth													
		Petrosal		Parscochlearis		Tegmentympani		Parsmastoidea		Cochlearcanal					Semicircularcanal angles			Semicircularcanal length			Semicircularcanal radius		
		Length	Width	Length	Width	Length	Width	Length	Width	Stapedial ratio	Cochlear coiling	Cochlear ratio	Length	Relative volume	lateral-anterior	lateral-posterior	Angle anterior-posterior	Anterior	Lateral	Posterior	Anterior	Lateral	Posterior
Sirenia	CBI-1-542	18	14	10	8	14	5	12	6	1,95	900°	0,67	24,50	62%	71°	88°	58°	6,17	6,76	6,35	1,82	1,89	1,70
	*Prorastomus*	37	39	21	20	12	30	22	24	1,54	550°	0,34	15,80	50%	85°	96°	88°	10,35	12,10	11,42	2,84	3,01	2,96
	*Dugong*	50	42	32	22	36	23	22	32	NA	514°	0,57	22,08	NA	78°	86°	103°	8,28	8,22	7,10	2,85	2,57	2,41
	*Trichechus*	NA	NA	NA	NA	NA	NA	NA	NA	1,60	407°	0,55	22,46	71%	52°	78°	85°	17,31	14,20	16,53	4,30	4,46	3,54
Proboscidea	*Elephas*	NA	NA	NA	NA	NA	NA	NA	NA	1,59	793°	0,55	38,10	26%	88°	58°	63°	18,90	10,15	14,36	4,19	2,58	3,73
Hyracoidea	*Procavia*	12	11	6	5	7	3	3	5	1,72	1183°	0,77	11,03	44%	85°	90°	100°	3,92	2,74	3,65	1,01	0,72	0,99

## Results

Order Sirenia; Family, Gen. and sp. Indet.

Locality: Djebel Chambi, late Ypresian to early Lutetian.

### Petrosal, Ventral Face (Fig. 1A, B)

CBI-1-542 is slightly more than half the size of the petrosal of *Prorastomus* (Table 1). The dorsal part of the *promontorium* and the region of the *fossa subarcuata* are broken. The ventral face of the *pars cochlearis* is well preserved. It is almost square in ventral view. Its surface is smooth. There are no sulci for the stapedial or the transpromontorial arteries. On the anteromedial face of the *pars cochlearis*, there is an articulation facet, maybe for the squamosal. As in *Prorastomus*, the *pars cochlearis* does not bear any evidence of a developed epitympanic wing. Such an epitympanic wing is present in *Eosiren*, *Eotheroides*, *Protosiren*, the undescribed protosirenid from Libya and extant sea-cows. The *promontorium* is a cone-shaped bulge. The *fenestra cochleae* (round window) opens caudally and very medially with respect to the *fenestra vestibuli* (oval window). The dorsal boundary of the *fenestra cochleae* was not preserved, which makes it impossible to see whether or not it was merged with the *canaliculus cochleae*. These two apertures are well separated by the *septum metacochleare* in *Prorastomus* [7], [16]. In more derived sea-cows in which this character is observable (*Protosiren*, the undescribed protosirenid from Libya and *Eosiren*), the *fenestra cochleae* is separated from the *fenestra vestibuli* by a thick *crista interfenestralis* [9], [17]. The *fenestra vestibuli* is strongly oval, when in *Prorastomus* and *Trichechus* it is rounded (Table 1). The *pars cochlearis* is separated laterally from the *tegmen tympani* (*pars temporalis*) by the *sulcus facialis*. It is wide and shallow. It ends rostrally in the secondary (tympanic) facial foramen, which is large. The *canalis fallopi* (facial canal) is short, leading dorsally to the facial foramen. Rostrally, it is bounded by a thick bony septum called the *commissura suprafacialis*. There is no pseudotympanic facial foramen, unlike what can be seen in *Prorastomus* and *Dugong* [6], [18]. In trichechids, the absence of a commissura suprafacialis to close the pseudotympanic facial foramen laterally precludes its presence. In other Paleogene sea-cows, its presence is impossible to ascertain. The tympanohyal process is short and robust. It bears a small fractured area which probably used to receive the posterior crus of the tympanic. The stapedial muscle fossa (*fossa muscularis minor*) is round and deep, as in *Prorastomus* only. In *Eosiren*, *Eotheroides*, *Protosiren* and extant sea-cows, the stapedial muscle fossa is shallower and its boundaries are unclear, especially medially. The *tegmen tympani* forms a reniform bulge on the lateral side of the petrosal in ventral view, as in *Eosiren*, *Eotheroides*, *Protosiren* and extant sea-cows [19] (in *Prorastomus* it is a little more squared [7]). However, it is not as inflated as in other sirenians, and its size is equivalent to the *pars cochlearis* and the *pars mastoidea* (fig.1; table1). The *tegmen tympani* is bounded mediodorsally by a deep longitudinal sulcus maybe for the *sinus petrosus superius*. The caudal-most extremity of the *tegmen tympani* bears a small fractured area on its caudal margin that probably corresponds to the articulation of the anterior crus of the ectotympanic. It suggests that the ectotympanic was probably fused with the petrosal. The rostral margin of the *tegmen tympani* does not bear any trace of articulation with the alisphenoid, unlike what can be observed in *Prorastomus* only [7]. The epitympanic recess (*recessus epitympanicus*), which received the incudo-mallear articulation, is a shallow depression on the lateral margin of the *tegmen tympani*. This depression is deeper in *Prorastomus*, *Eosiren*, *Eotheroides*, *Protosiren* and extant sea-cows.

### Petrosal, Dorsal Face (Fig. 1C)

**Figure 2:**
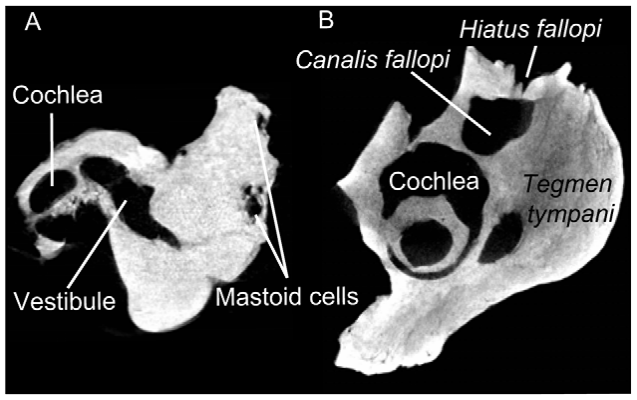
CT-radiography of CBI-1-542. A: slide at the level of the *pars mastoidea* showing cancellous cells in the mastoid apophysis; B: slide at the level of the *hiatus fallopi* showing the communication between the *hiatus fallopi* and the *canalis fallopi*.

Cerebellar face. Most of the dorsal part of the petrosal is broken. The inner auditory meatus is a large aperture on the cerebellar face of the petrosal. It is bounded rostrolaterally by the *commissura suprafacialis*. It is divided in two main acoustic foramina (*foramen acusticum anterius* and *posterius*) for the vestibulocochlear nerve (nerve VIII) (see Fig.1D for more details). As in *Prorastomus*, these acoustic foramina are only separated by a thin osseous septum, whereas in *Eosiren*, the undescribed protosirenid from libya and extant sea-cows -which are the only one in which this character can be observed- the facial foramen and the *foramen acousticum posterius* are well separated by a thick septum. The dorsal part of the *promontorium* is broken. As observed on the scans of *Prorastomus* [16], *Protosiren* [20], *Trichechus* [21] and *Dugong*, it is made of dense and thick (pachyosteosclerotic) bone. The *acqueductus vestibuli* (endolymphatic foramen) opens caudolaterally in a narrow fissure. The posterior-most part of the periotic is formed by the mastoid apophysis (*pars mastoidea*). This apophysis is bulbous and small with respect to the strongly inflated and square mastoid apophysis of *Prorastomus*, *Eosiren*, *Eotheroides*, *Protosiren* and extant sea-cows. There is no *processus fonticulus*. The mastoid consists of cancellous bone as demonstrated by CT-scanning (Fig.2A). In contrast, the *pars mastoidea* is made of dense (osteosclerotic) bone in at least *Prorastomus*, *Protosiren* [20] and extant sirenians.

### Cerebral Face

A dorsoventrally-oriented furrow excavates the cerebellar face of the petrosal and divides the *commissura suprafacialis* into a dorsal and ventral part. CT-scanning reveals that small foramina enter this furrow and lead to the lumen of the *canalis fallopi* (Fig. 2B). Thus this furrow is homologous to the *hiatus fallopi* which corresponds to the exit of the greater petrosal nerve (or vidian ramus of the facial nerve). A *hiatus fallopi* is absent in *Prorastomus* and in extant sirenians [18]. This character cannot be ascertained in other Paleogene sea-cows.

### Bony Labyrinth (Fig. 3)

**Figure 3:**
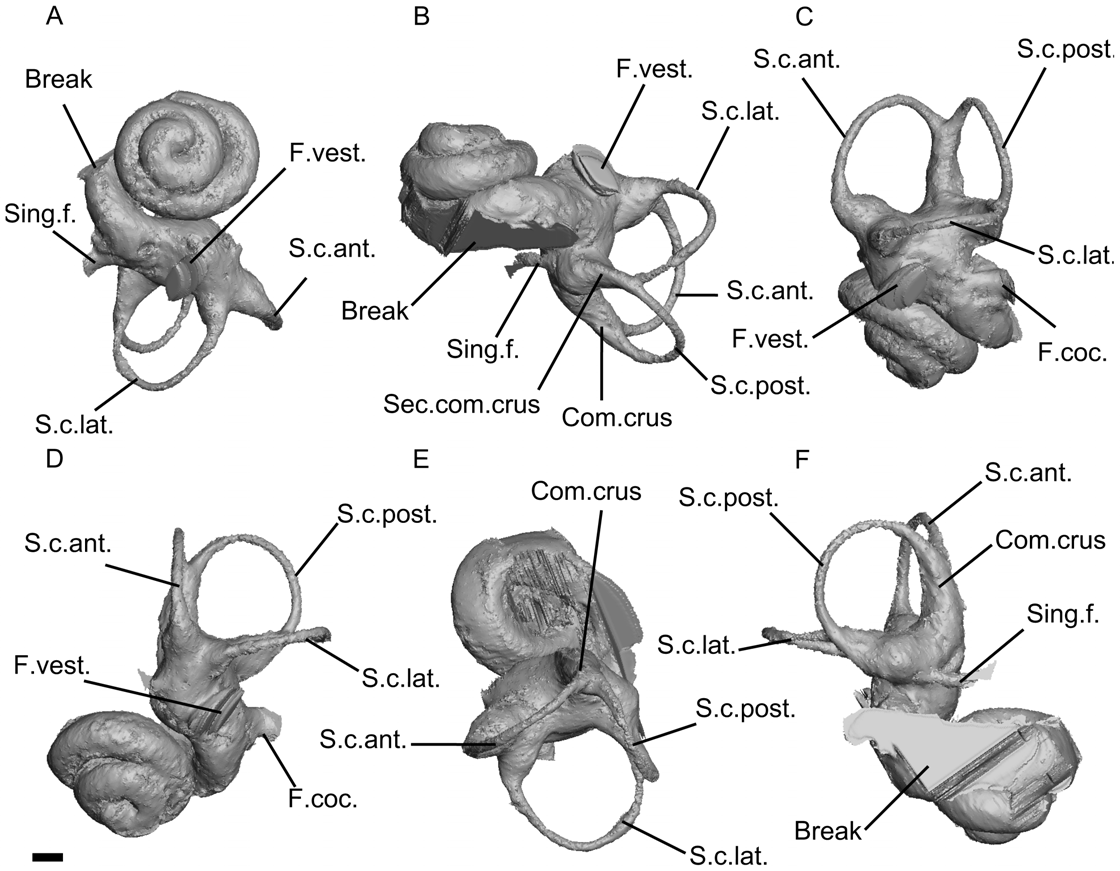
CT reconstruction of the bony labyrinth of the sirenian from Chambi. A, ventral view (apical view of the cochlear canal); B, posterolateral view (profile view of the cochlear canal); C, lateral view; D, anterior view; E, dorsal view; F, posterior view. Scale bar = 1mm. Legend: Acq.vest.: bony channel of the *acqueductus vestibuli*; Break: broken area of the bony labyrinth; Can.coc.: bony channel of the *canaliculus cochleae*; Com.crus: common crus; F.vest.: bony channel of the *fenestra vestibuli*; F.coc.: bony channel of the *fenestra cochleae*; S.c.ant.: anterior semicircular canal; S.c.lat.: lateral semicircular canal; S.c.post.: posterior semicircular canal; Sec.com.crus: secondary common crus; Sing.f.: bony channel of the singular foramen.

**Figure 4:**
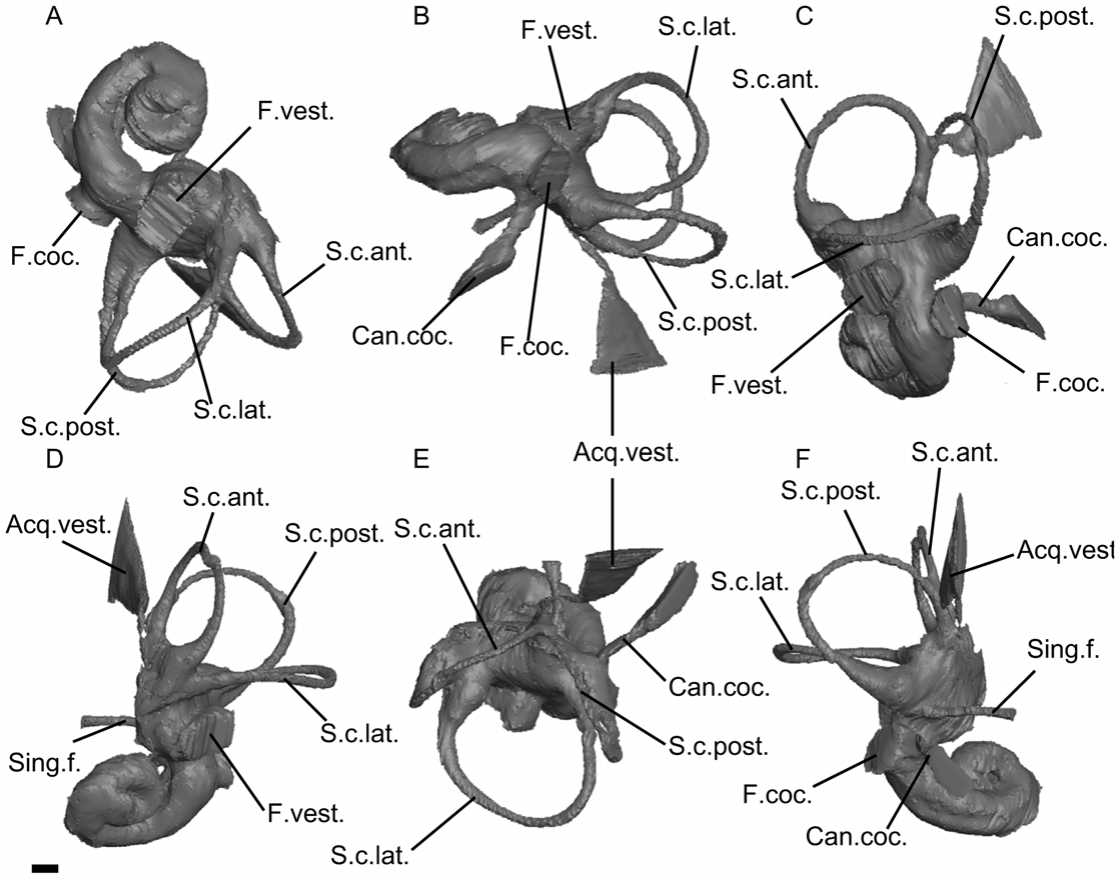
CT reconstruction of the bony labyrinth of *Prorastomus* (mirrored). A, ventral view (apical view of the cochlear canal); B, posterolateral view (profile view of the cochlear canal); C, lateral view; D, anterior view; E, dorsal view; F, posterior view. Scale bar = 1mm. Legend: Acq.vest.: bony channel of the *acqueductus vestibuli*; Break: broken area of the bony labyrinth; Can.coc.: bony channel of the *canaliculus cochleae*; Com.crus: common crus; F.coc.: bony channel of the *fenestra cochleae*; F.vest.: bony channel of the *fenestra vestibuli*; S.c.ant.: anterior semicircular canal; S.c.lat.: lateral semicircular canal; S.c.post.: posterior semicircular canal; Sec.com.crus: secondary common crus; Sing.f.: bony channel of the singular foramen.

The digitally-reconstructed cast of the bony labyrinth is almost complete. The cochlear canal makes 2.5 turns (900°) which is nearly twice the value of the cochlear coiling in extant sirenians (Table 1). However, as in *Prorastomus* and extant sirenians, the second turn of the cochlear canal does not overlap the basal turn in ventral (apical) view. When viewed in profile (Fig. 3B), the cochlear spiral is high and conical, whereas it is planispiral in *Prorastomus* (Fig. 4B) and extant sirenians [21]. There is no bony *lamina secundaria*. The volume of the preserved part of the cochlear canal constitutes 62% of the total volume of the bony labyrinth. This neglected value is intermediate between *Prorastomus* and the extant manatee (Table 1). The region of the perilymphatic sac is hidden and thus does not allow any observation of the degree of fusion of the perilymphatic canal with the canal leading to the *fenestra cochleae*. In *Prorastomus*, these canals are well separated, except at their base where they merge (Fig. 4B). On CBI-1-542, the *fenestra vestibuli* opens ventrally, at the base of the cochlear canal. As in other sirenians examined, the *recessus sphericus* and the *recessus ellipticus* are not well separated. However, the ampullae are voluminous and well differentiated, as in *Prorastomus*, whereas they are indistinct in modern sirenians [21]. The cast of the vestibule is smaller than the cochlear canal. The semicircular canals form three cylindrical bony channels which do not display any major undulation. On the other hand, *Prorastomus* displays a gently undulate lateral canal (Fig. 4C). On CBI-1-542, the values of the angles measured between the planes of each canal are close to 90°. The semicircular canals are as high as they are wide. Their radius is about the same for each canal, as in *Prorastomus* and modern sirenians (Table 1). However, the lateral canal is slightly greater in terms of radius and length, as in *Prorastomus* and *Trichechus* (Table 1). The lumina of the lateral and posterior semicircular canals merge together and form a short secondary common crus (*crus communis secundaria*) which enters the posterior ampulla (Fig. 3B). This secondary common crus is absent in *Prorastomus* and extant sirenians.

## Discussion

### Relationship to Sirenia

**Figure 5:**
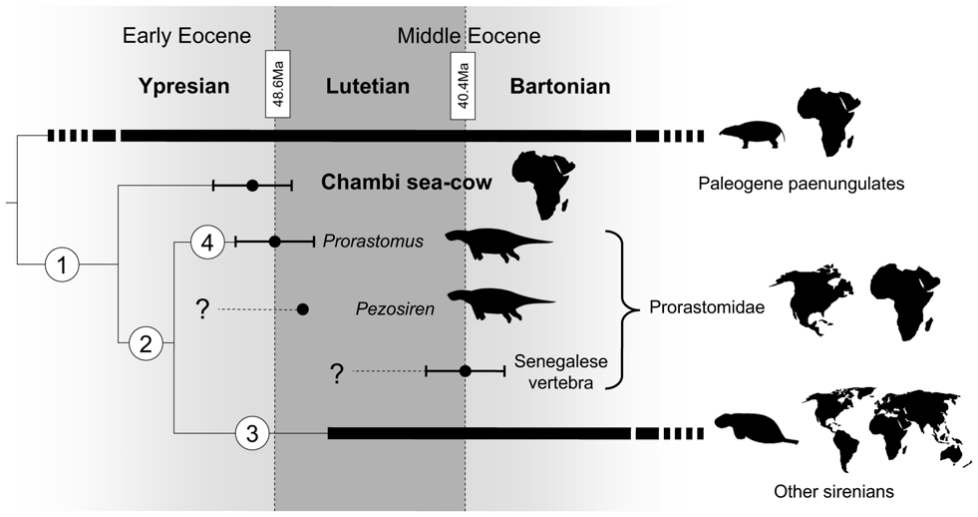
Simplified result of the cladistic analysis on sirenian petrosal and bony labyrinth characters (see also S1). Strict consensus of 2975 trees. Tree length: 57; Consistency index (CI): 0.60; Retention index (RI):0.81; Homoplasy index (HI): 0.50; Rescaled consistency index (RC): 0.48. Bold lines represent fossil record. Geographic ranges of the sirenian from Chambi, prorastomids and other sirenians after refs. 3, 5, 12. Shared derived traits at nodes: Node 1: pachyosteosclerotic promontorium and *tegmen tympani* (3(1)); reniform *tegmen tympani* (11(1)); cochlear canal more voluminous than the vestibule (23(1), homoplasic, CI = 0.5); semicircular canals of approximately the same radius (25(1)); lateral canal larger than other canals (26(1)). Node 2: reduced stapedial ratio (round *fenestra vestibuli*) (5(1), homoplasic CI = 0.33); *Hiatus fallopi* absent (7(0), Homoplasic, CI = 0.33); Pseudotympanic facial foramen present (15(1)); inflated, squared (17(2)) and dense (19(1)) mastoid apophysis. Node 3: merged *canaliculus cochleae* and *fenestra cochleae* (4(1), homoplasic, CI = 0.33); weakly defined stapedial muscle fossa (10(1)); large and deep epitympanic recess (13(1)); developed epitympanic wing (16(1), homoplasic, CI = 0.2); Thick *crista falciformis* widely separating the acoustic foramina (20(1), homoplasic, CI = 0.50). Node 4: tegmen process present (12(1), homoplasic, CI = 0.5).

In order to ascertain the attribution of CBI-1-542, we performed a cladistic analysis on a variety of paenungulate taxa including sirenians, hyraxes and proboscideans (see S1). This analysis supports the assignment of CBI-1-542 to a stem sirenian (Fig. 5, node 1). Regarding the highly autapomorphic condition of the ear region in sirenians, the allocation of CBI-1-542 to the Sirenia is based on a suite of derived anatomical ear details that are otherwise found in sirenians [see 16–24]. As in Sirenia, CBI-1-542 displays a *pars petrosa* and a *tegmen tympani* made of pachyosteosclerotic bone (3(1)), an inflated and reniform *tegmen tympani* (11(1)), a bony vestibule clearly less voluminous than the cochlear canal (23(1); CI = 0.5), a bony labyrinth having its three semicircular canals of similar size (25(1)), and a lateral semicircular canal slightly larger than the other ones (26(1)). Some of these characters (inflated *tegmen tympani*, osteosclerosis, semicircular canals of about the same radius, and reduced vestibule) are also present in cetaceans of modern aspect and are probably linked to the aquatic behaviour shared by cetaceans and sea cows, which are the only fully marine mammals [16], [21], [23]–[26]. Nevertheless, all petrosals known for Archaeoceti bear a strong and voluminous mastoid apophysis, which articulates with the cranium [26]. This apophysis is absent on CBI-1-542. Such a state, called amastoidy, seems to be a shared derived trait of Paenungulata [22] (character 17 in this analysis, see S1). Moreover, in archaeocetes, the facial foramen is very small and located close to the *fenestra vestibuli*, while on CBI-1-542, the foramen is a very large hole located more rostrally on the petrosal. It also differs from the petrosal of archaeocetes in lacking the anterior process of the *tegmen tympani*, in showing no vascular groove and notch for the superior ramus of the stapedial artery, in having no development of a deep fossa for the head of the malleus, and in lacking the sigmoid process (Fig. 1).

### Comparison with Other African Sirenians and *Prorastomus*: Paleobiogeographical Implications

Among the taxa of sea cows found in the Paleogene of Africa, the petrosal morphology is only available for a few genera: *Eosiren* (*E. abeli* from the middle Eocene of the Djebel Mokattam (Egypt)) [27], [28], *Eotheroides* (*E. lambondrano* from the middle Eocene of the Mahajanga Basin (Madagascar) and *E.* n.sp. from the late Eocene of Wadi Al Hitan (Egypt)) [29], [30], Protosirenidae n.gen. n.sp. from the middle Eocene of Bu el Haderait (Libya)) [9] and *Protosiren* (*P. fraasi* from the middle Eocene of the Djebel Mokattam (Egypt) and *P.* sp. from North America) [13], [19]. The petrosal from Chambi differs from that of *Protosiren* by the presence of a *commissura suprafacialis* and the absence of a developed *processus fonticulus* on the mastoid apophysis (however, the *processus fonticulus* is also absent on the specimen found in North America [13]). It differs from the petrosal of the Libyan protosirenid by the presence of a thick *commissura suprafacialis* and by the dimensions of the *tegmen tympani* which nearly equal the size of the mastoid apophysis, whereas it is significantly larger in the Libyan protosirenid. It differs from the petrosal of *Eotheroides* in the presence of a well-defined and circular stapedial muscle fossa, whereas the boundaries of this fossa are faint in *Eotheroides*, and in the rectangular instead of triangular anterior margin of the *tegmen tympani*. The petrosal from Chambi more closely resembles that of *Eosiren*: they share a thick *commissura suprafacialis*, a poorly developed *tegmen tympani* and a reduced mastoid apophysis. However, these characters are plesiomorphic among sirenians [9]. Moreover, CBI-1-542 differs from the petrosal of *Eosiren* in the presence of a well-defined and circular stapedial muscle fossa, the absence of a strongly developed and finger-like tympanohyal process, the small distance between the *foramen acusticum posterius* and the *foramen acusticum anterius*, and by the absence of a developed *processus fonticulus* on the mastoid apophysis. Finally, given that CBI-1-542 is smaller than the petrosal of *Prorastomus* which is the smallest sirenian known [7], the petrosal of the sirenian from Chambi can be distinguished by its even smaller size (Table 1).

According to the cladistic analysis performed here, we can ascertain that the morphology of CBI-1-542 is more primitive than that of *Prorastomus* and younger sirenians. First, the petrosal of *Prorastomus* is characterized by the presence of a square process on the *tegmen tympani* called the tegmen process [20] and a fusion of the *tegmen tympani* with the alisphenoid (Fig. 1E), which is absent in all other sirenians [7], [9] including that of Chambi (Fig. 5, node 4, character (12(1))). The petrosal from Chambi also differs in the morphology of its *pars mastoidea*, which is very small with respect to that of *Prorastomus* and those of extant and Paleogene genera (Table 1). In CBI-1-542, the mastoid primitively forms a short process, as in other paenungulates [22], while in *Prorastomus* and other sirenians examined, it is a prominent squared process (Fig. 5, node 2, character 17(2)). It was already suggested that earliest sirenians may not have displayed such a hypertrophy of the mastoid process and that their mastoid would not have been exposed on the occipital face of the skull, unlike what occurs in most sirenians [22]. In addition, CT-scanning allows the observation of cancellous bone within the mastoid apophysis of CBI-1-542 (Fig. 2A), while in *Prorastomus* and younger sirenians, this internal bone structure is dense (osteosclerotic) (Fig. 5, node 2, character 19(1)). Cancellous cells are primitively present in the *pars mastoidea* of most mammals. Furthermore, the sirenian from Chambi appears clearly more primitive than *Prorastomus* because it possesses a relictual *hiatus fallopi* (Fig. 2B). This foramen is usually present in ungulates [31] but is lost in *Prorastomus* and the other species studied (Fig. 5, node 2, character 7(0), CI = 0.33). In fact, in *Prorastomus* the *canalis fallopi* is ventrally opened between the *hiatus fallopi* and the secondary facial foramen. It does not form a pipe but a deep sulcus (Fig. 1D). The *hiatus fallopi* is also absent in the embrithopod *Arsinoitherium* [20] and is sporadically present in some Pleistocene proboscideans [32]. Given that condylarthrans, modern hyracoids and the early proboscidean *Phosphatherium* possess a true aperture for the *hiatus fallopi* [31], [33], this structure must has been lost convergently among paenungulates. The Chambi taxon also differ from the clade gathering other sirenians by the absence of a pseudotympanic foramen (Fig. 5, node 2, character 15(1), CI = 0.50). However this character should be regarded cautiously because many taxa are not coded for this character (see S1). Finally, the *fenestra vestibuli* of CBI-1-542 displays a stapedial ratio of 1.95, corresponding to an oval shape. This value is significantly greater than that measured in extant sirenians, proboscideans and *Arsinoitherium* (1.6 after ref. 21), which corresponds to a rather rounded *fenestra vestibuli*. However, the value of this ratio is 1.72 in the living hyracoid *Procavia*, 1.82 in the Eocene proboscidean *Numidotherium* [34] and 1.8 to 1.9 in some Pleistocene elephantoids [32]. Given that a stapedial ratio exceeding 1.8 seems to be plesiomorphic for eutherian mammals [21], [35], it seems that the reduction of the stapedial ratio among Paenungulata occurred convergently in most orders and that the sirenian from Chambi displayed the primitive state for this character (character 6(1)). However we can’t be more precise since the polarity of this character is ambiguous in the cladistic analysis. The bony labyrinth of CBI-1-542 can be distinguished from those of *Prorastomus* and later sirenians by its great degree of cochlear coiling (2.5 turns). The cochlear canal of CBI-1-542, especially regarding its height and coiling, is more closely related to the one found in living hyracoids and the Eocene/Oligocene proboscidean *Moeritherium*, which make two turns or more and have an aspect ratio of 0.72 [36]. However, the cochlear canal of *Numidotherium* is low and planispiral [36], which makes the polarity of this character ambiguous for paenungulates. Finally, the bony vestibule of CBI-1-542 displays a secondary common crus where the posterior limb of the lateral canal and the ampullar limb of the posterior canal merge together (Fig. 3B). This character is primitive for Theria [21], [36] and seemingly for Afrotheria because it is also present in *Orycteropus*, *Numidotherium* and the early macroscelidid *Chambius* [37]. Conversely, a secondary common crus is absent in *Prorastomus* and extant sirenians (Fig. 4). In sum, it appears that the sirenian from Chambi, which is represented by the unique CBI-1-542 petrosal, was clearly more primitive in several anatomical details of the ear than *Prorastomus* and other Paleogene sirenians. Such a primitiveness of the ear region emphasizes the basal position of this new African taxon within the Sirenia clade, and as such testifies to the great antiquity of this group in Africa (Fig. 5). Accordingly, this discovery supports the African origin of the Sirenia clade, and hence helped to elucidate the biogeographical paradox of their origin.

### Aquatic Lifestyle

As stated above, some morphological characteristics of the middle and osseous inner ear of the sirenian from Chambi display noticeable convergences with those of cetaceans. For example, pachyosteosclerosis of the middle ear is correlated with underwater hearing [16], [23] and, in strictly aquatic mammals, semicircular canals are smaller in terms of radius of curvature, presumably because of the decrease in neck and head mobility correlatively to the increase in body hydrodynamism [25]. These traits advocate for an aquatic lifestyle. An aquatic lifestyle for “prorastomids” is well documented by several morpho-functional clues (e.g., dorsal opening of the nares, lack of paranasal sinuses, pachyosteosclerotic ribs), and other authors have emphasized that they were already adapted to life in water, in a way similar to the archaeocetes *Ambulocetus* and *Rodhocetus* [1], [3], [8]. Court [16] also pointed out that in *Prorastomus*, the *pars cochlearis* is made of osteosclerotic bone, a character correlated with underwater hearing [16], [23]. The *pars cochlearis* of CBI-1-542 is also made of dense bone, which implies that the Chambi sea cow was probably able to hear underwater. Moreover, it is now well established that in marine mammals the morphology of the vestibule is closely related to aquatic locomotion [25]. Aquatic mammals such as sirenians usually display smaller semicircular canal radii than their terrestrial counterparts, as well as a relatively reduced vestibule (in terms of volume) with respect to the cochlear canal [21], [23]-[25]. The vestibular morphology of CBI-1-542 closely resembles that of *Prorastomus* and extant sirenians, notably regarding the relatively small semicircular canal radii and the reduced volume of the vestibule (Table 1). Considering these similarities, it might be expected that the sirenian from Chambi was already well adapted to life in water.

### Paleoecology and Paleoenvironment of Early Sea Cows

For a terrestrial mammal, transition to a marine or freshwater environment necessitates dramatic physiological adaptations (e.g., osmoregulation) [38], [39]. *Prorastomus* and *Pezosiren* were found in coastal and estuarine deposits, which correspond to brackish waters [7], [8]. Other paleontological clues suggest they were probably already adapted to marine environments, but given the greater diversity of aquatic angiosperms in freshwater, one cannot exclude occasional freshwater foraging [1], [6], [7]. In contrast, the vertebrate locality of Djebel Chambi in Tunisia is a bed of lacustrine limestone, which should correspond to freshwater deposits [14]. The presence of charophytes and amphibian remains in the locality supports this paleoenvironmental reconstruction [14]. Noteworthily, the discovery of a tooth of an electric ray (Chondrichthyes, Torpediniformes) in this fauna suggests occasional connections with sea water (S2). Likewise, some occasional connections to the Tethys Sea are attested by the presence of marine deposits with tidal influence (sigmoidal tidal bundles in limestones) in the uppermost part of the stratigraphic series. Based on isotopic analysis, it has been proposed that sirenians “took a more direct route than archeocetes into the marine environment” [38], [39], which means that they adapted to life in the sea without first passing through a more freshwater habitat. However, these authors emphasized the unfortunate absence of “prorastomids” in their samples. The opposing hypothesis is that sirenians were adapted first to freshwater or were at least euryhaline, mainly because fresh water provides a greater diversity of aquatic angiosperms than the sea does [7]. The discovery of CBI-1-542 in lacustrine calcareous deposits gives support to the scenario involving a small freshwater or euryhaline archaic sirenian.

### Concluding Remarks

**Figure 6:**
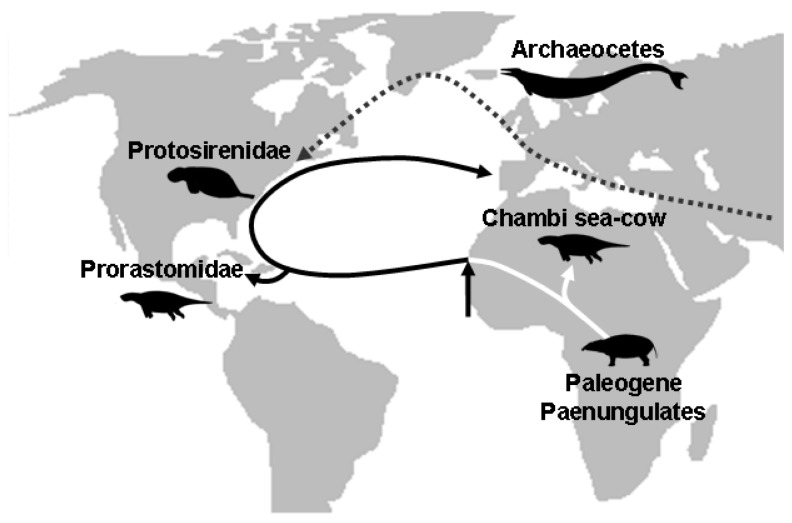
Hypothetical dispersal routes of stem sirenians (solid line) and archeocetes (dotted line) during the Early and early Middle Eocene, after 46. The arrow indicates the transition from freshwater (white line) to sea-water sea-cows (black line).

The hypothesis of an amphibious ancestry of sea-cows, elephants and their extinct relatives has been heavily documented so far. In Proboscidea, isotopic analysis suggest that stem taxa (*Moeritherium* and *Barytherium*) may have feed on freshwater plants and lived in freshwater swamps [40]. This assertion is supported by the study of developmental growth in modern elephants which has shown that they display some characters involving a past adaptation to life in water (e.g. prolonged persistence of nephrostomes in the mesonephros) [41]. The Desmostylia were also amphibious and probably used to live in freshwater or estuarine environment [42]. On the basis of skeletal anatomy, other fossil relatives of sirenians and elephants were also supposed to spend most of their time in fresh or estuarine waters (i.e. *Arsinoitherium*, *Numidotherium*, the Anthracobunidae) and taken together, all these data point out that the last common ancestor of the Sirenia and Proboscidea probably lived in swamps or mangroves [43-45, but see 38]. Perhaps freshwater plants eating has limited the distribution of archaic sirenians before their radiation in the late Early Eocene. It has been supposed that archaeocetes dispersed to the New World travelling along the west cost of Europe [46]. However, in the case of sirenians a dispersal route directly through the Atlantic sea appears more likely (Fig.6) because no remains of stem sirenians were found in the rich Eocene marine deposits of Europe prior to the middle Eocene [46], [47]. Hence, this first wave of migrants (from which *Prorastomus* is a descendant) was probably adapted to salt waters. Under this assumption, the transition from fresh to sea waters may have been an important landmark of sirenians evolutionary history, allowing them new dispersal capacities. Given the late-Early to Early-middle Eocene age of *Prorastomus* and the Chambi taxon, the minimum age of this transition is early Eocene. Because the first marine sea-cows may have already feed on seagrasses [13], this age should not prior the Cretaceous period, the time when seagrass beds raised in the Tethys Sea [48].

## Materials and Methods

CBI-1-542 is an almost complete left petrosal bone, lacking a part of the dorsal face. It is housed at the collections of the *Office National des Mines de Tunis*, Tunisia. The scan was made at the MRI Micro-CT imaging station *Skyscan 1076* (Montpellier, France) with a resolution of 18.8 µm (CBI-1-542). Although the external morphology of the petrosal of *Prorastomus* has already been briefly described [7], [16], its petrosal and bony labyrinth have been reconstructed here too and are extensively figured for the purpose of comparison (Fig. 1D, E, F, Fig. 4). The basicranium of the type specimen (BMNH 44897) was scanned at the British Museum of Natural History (London, UK) with a resolution of 79 µm. Comparative samples were scanned at the MRI Micro-CT imaging station *Skyscan 1076* (Montpellier, France) with a resolution of 36.7 µm (*Procavia*, *Dugong*) and at the University Hospital Center of Montpellier *MultiDetector CT scanner Lightspeed VCT* (Lapeyronie Hospital, Montpellier, France) with a resolution of 350µm (*Elephas*). Image segmentation and 3D reconstructions of petrosals and bony labyrinths were done using Avizo 6.3 (VSG) software. Measurements of bony labyrinths (Table 1) were taken using Avizo 6.3 software. A summary of the measurement protocol is available in S3. In order to allow comparison with in-situ material, petrosals are described in their hypothetical in-situ orientation. On the contrary, orientation of the bony labyrinth was simplified: the anterior view of the bony labyrinth is orthogonal to the plane of the anterior duct, the posterior view is orthogonal to the plane of the posterior duct, and the lateral view is in line with the plane of the lateral duct. In addition to these taxa (*Prorastomus*, *Dugong*, *Elephas* and *Procavia*), CBI-1-542 was also compared with the extant manatee *Trichechus* [17], [21] and all Paleogene sirenians of Africa for which the petrosal morphology is published. The following bibliographic references were used for the comparative description: *Eosiren* [27], [28], *Eotheroides* [29], [30], Protosirenidae n.gen. n.sp. [9] and *Protosiren* [13], [20]. Details about the cladistic analysis are available online in the S1. The character matrix is available in S4. Requests for material and scans should be addressed to Dr. Tabuce (rodolphe.tabuce@univ-montp2.fr).

## Supporting Information

Information S1 **Cladistic analysis method.**
(DOC)

Information S2 **Ray tooth description.**
(DOC)

Information S3 **Measurement protocol.**
(DOC)

Information S4 **Character matrix for the cladistic analysis (nexus format).**
(NEX)
