# Supplementary material for: Cranial Remain from Tunisia Provides New Clues for the Origin and Evolution of Sirenia (Mammalia, Afrotheria) in Africa
Source: PLoS One. 2013 Jan 16;8(1):e54307. doi: 10.1371/journal.pone.0054307 (PMC3546994; doi:10.1371/journal.pone.0054307)
Supplement: Information S1 — Cladistic analysis method. (DOC) [file pone.0054307.s001.doc]

S1. Cladistic analysis method

In order to test the assignment of CBI-1-542, we performed a cladistic analysis on a datamatrix 22 taxa and of 27 petrosal and bony labyrinth (when available) characters. We included in this analysis both genera of modern-day sirenians as well as most taxa of Paleogene sirenians for which the petrosal is known: *Prorastomus sirenoides* (this study), *Eosiren* (*E. abeli* from the middle Eocene of the Djebel Mokattam (Egypt)) [1, 2], *Eotheroides* (*E. lambondrano* from the middle Eocene of the Mahajanga Basin (Madagascar) and *E.* n.sp. from the late Eocene of Wadi Al Hitan (Egypt)) [3, 4], Protosirenidae n.gen. n.sp. from the middle Eocene of Bu el Haderait (Libya)) [5] and *Protosiren* (*P. fraasi* from the middle Eocene of the Djebel Mokattam (Egypt) and *?P.* sp. from North America) [6, 7]. The stem sirenian *Pezosiren* was omitted because its petrosal morphology is to date not published. The taxon sampling also comprises the Paleogene paenungulates *Phosphatherium* [8], *Numidotherium* [9, 10, 1], *Moeritherium* [12] and *Arsinoitherium* [13] (bony labyrinth coded according to personal observations). We code the modern taxon Elephantoidea based on observation of *Elephas maximus* and the published study of Ekdale [14]. Studies dealing with the morphology of the ear region of hyracoids are scarce, and with the exception of Thewissen and Simons [15] who briefly described the petrosal and bullar morphology of *Megalohyrax eocaenus*, no careful surveys of the petrosal of Paleogene Hyracoidea were conducted. As a consequence Hyracoidea are represented in this analysis by this sole Paleogene species and the modern day Cape hyrax *Procavia capensis*. In the same manner, the petrosal morphology of desmostylians and anthracobunids has never been described and as such these taxa have not been considered in this analysis. The analysis was rooted using the petrosal and bony labyrinth morphology of the Cretaceous eutherian mammal *Kulbeckia kulbecke* [16, 17, 18]. *Orycteropus afer* and several Condylarthrans were also used as outgroups: *Meniscotherium* [19, 20], *Hyopsodus* [11, 21], *Phenacodus* [22], *Arctocyon* [23], *Pleuraspidotherium* [23, 24]. The analysis was performed using PAUP4b10 [25], with a heuristic search. All characters were treated as unordered and equally weighted.

The result of the cladistic analysis is a well resolved strict consensus of 2975 trees (trees length=57) (supp.fig.1). The value of the retention index is very good (RI=0.81). The consistency index (CI) equals 0.60, the rescaled consistency index equals 0.48 and the homoplasy index equals 0.50. This result supports the monophyly of Sirenia and the attribution of CBI-1-542 to a species belonging to this order (see text). It also supports Paenungulata but not Proboscidea and Hyracoidea, may be because of our poor knowledge of the ear anatomy of *Megalohyrax*.


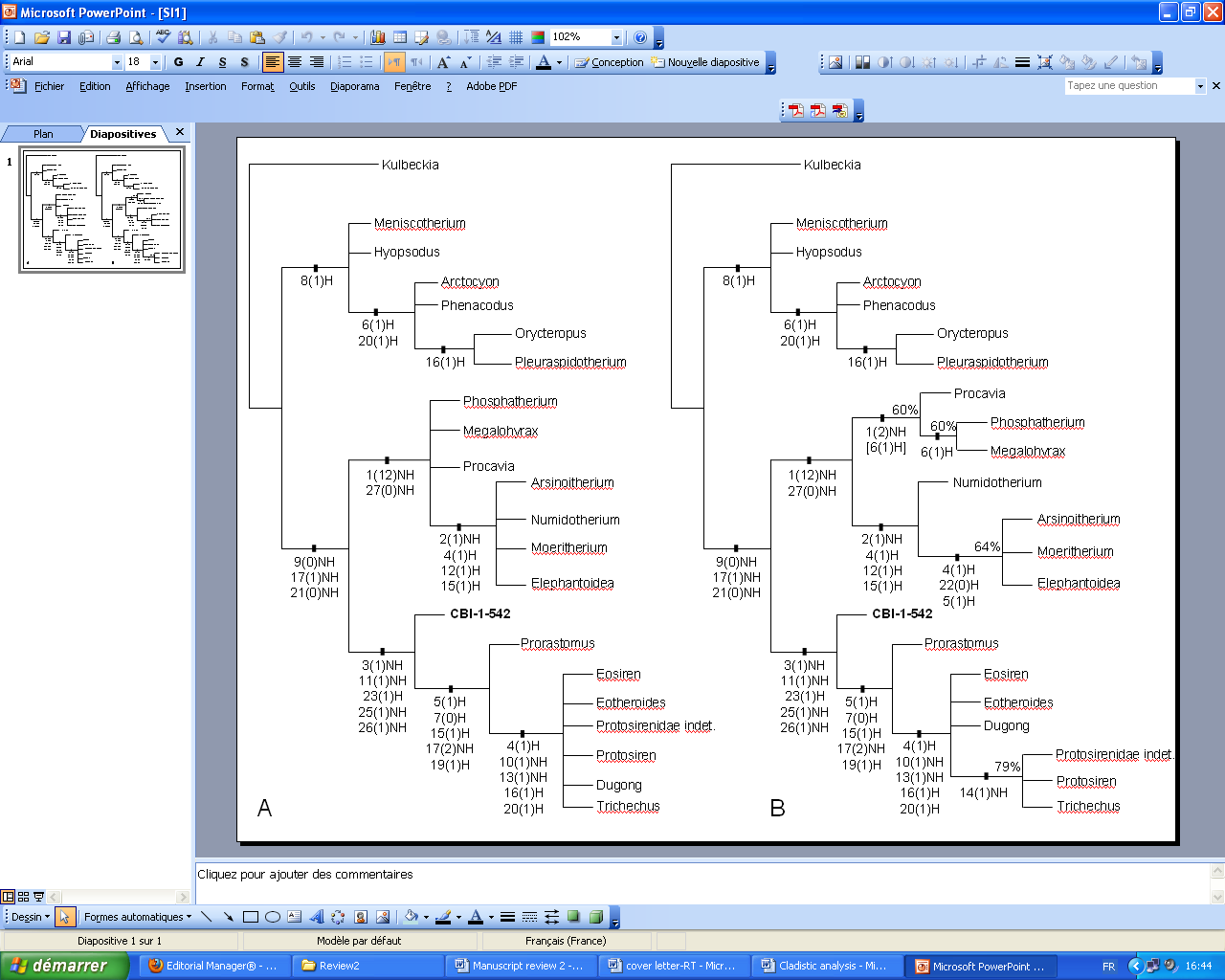


Fig.1. Results of the cladistic analysis. A. Strict consensus tree; B. Majority-rule consensus.

References

1. Abel O (1913) Die eocanen Sirenen der Mittelmeerregion. Erster Teil: Der Schädel von *Eotherium aegyptiacum*. Palaeontographica 59: 289-360.

2. Sickenberg O (1934) Beiträge zur Kenntnis tertiäre Sirenen. Mém Mus Roy Hist Nat Belgique 63: 1-352.

3. Samonds KE, Zalmout IS, Irwin MT, Krause DW, Rogers RR, et al. (2009) *Eotheroides lambondrano*, new Middle Eocene seacow (Mammalia, Sirenia) from the Mahajanga Basin, Northwestern Madagascar. J Vertebr Paleontol 29: 1233-1243.

4. Zalmout IS (2008) Late Eocene Sea Cows (Mammalia, Sirenia) from Wadi Al Hitan in the Fayum Basin (Egypt). PhD dissertation. University of Michigan, Ann Arbor.

5. Sagne C (2001) La diversification des siréniens à l’Eocène (Sirenia Mammalia): étude morphologique et analyse phylogénétique du sirénien de Taulanne *Halitherium taulannense*. PhD dissertation. Muséum National d’Histoire Naturelle Paris.

6. Domning DP, Morgan GS, Ray CE (1982) North American Eocene sea cows (Mammalia Sirenia). Smithson Contrib Paleobiol 52: 1-69.

7. Gingerich PD, Domning DP, Blane CE, Uhen MD (1994) Cranial morphology of *Protosiren fraasi* (Mammalia, Sirenia) from the middle Eocene of Egypt: a new study using computed tomography. Contr. Mus. Pal. Univ. Michigan 29: 41-67.

8. Gheerbrant E, Sudre J, Tassy P, Amaghzaz M, Bouya B, et al. (2005) New data on *Phosphatherium escuilliei* (Mammalia Proboscidea) from the early Eocene of Morocco and its impact on the phylogeny of Proboscidea and lophodont ungulates (translated from French). Geodiversitas 27: 239-333.

9. Court N, Jaeger J-J (1991) Anatomy of the periotic bone in *Numidotherium koholense*, an example of parallel evolution in the inner ear of tethytheres. C R Acad Sci II, 312: 559-565.

10. Court N (1992) Cochlea anatomy of *Numidotherium koholense*: Auditory acuity in the oldest known proboscideans. Lethaia 25: 211-215 (doi: 101111/j1502-39311992tb01385x).

11. Benoit J, Orliac M, Tabuce R (2012) The petrosal of *Chambius* (Macroscelidea Afrotheria) from the Eocene of Djebel Chambi (Tunisia). J Syst Paleontol in press.

12. Court N (1994) The periotic of Moeritherium (Mammalia Proboscidea): homology or homoplasy in the ear region of Tethytheria McKenna 1975. Zool J Linn Soc 112: 13-28 (doi: 101111/j1096-36421994tb00310x).

13. Court N (1990) Periotic anatomy of *Arsinoitherium* (Mammalia Embrithopoda) and its phylogenetic implications. J Vertebr Paleontol 10: 170-182.

14. Ekdale EG (2011) Morphological variation in the ear region of Pleistocene Elephantimorpha (Mammalia Proboscidea) from central Texas. J Morphol 272: 452-464 (doi: 101002/jmor10924).

15. Thewissen JGM, Simons EL (2001) Skull of *Megalohyrax eocaenus* (Hyracoidea, Mammalia) from the Oligocene of Egypt. J Vertebr Paleontol 21: 98-106.

16. Ekdale EG, Archibald JD, Averianov AO (2004) Petrosal bones of placental mammals from the Late*.* Cretaceous Uzbekistan*.* Acta Palaeontol Pol 49: 161-176.

17. Ekdale EG (2009) Variation within the bony labyrinth of mammals. PhD dissertation. University of Texas Austin.

18. Ekdale EG, Timothy R (2011) Morphology and Variation within the Bony Labyrinth of Zhelestids (Mammalia, Eutheria) and Other Therian Mammals. J Vertebr Paleontol 31: 658-675 (doi: 10.1080/02724634.2011.557284).

19. Gazin CL (1965) A study of the early Tertiary condylarthran mammal *Meniscotherium*. Smith Misc Coll 149**:** 1-98.

20. Williamson TE, Lucas SG (1992) *Meniscotherium* (Mammalia, "Condylarthra") from the Paleocene - Eocene of Western North America. Bulletin of the New Mexico Museum of Natural History and Science 1: 1-54.

21. Gazin CL (1968) A Study of the Eocene Condylarthran Mammal *Hyopsodus*. Smith Misc Coll 153: 1-90.

22. Cifelli RL (1982) The petrosal structure of *Hyopsodus* with respect to that of some other ungulates and its phylogenetic implications. J Paleontol 56: 795-805.

23. Russell DE (1964) Les mammifères paléocènes d'Europe. Mém Mus Nat Hist Nat C 8: 1-324.

24. Ladevèze S, Missiaen P, Smith T (2010) First skull of *Orthaspidotherium edwardsi* (Mammalia, "Condylarthra") from the late Paleocene of Berru (France) and phylogenetic affinities of the enigmatic European family Pleuraspidotheriidae. J Vertebr Paleontol 30:1559-1578.

25. Swofford DL (2002) PAUP*. Phylogenetic Analysis Using Parsimony (and Other Methods). Version 4. Sinauer Associates, Sunderland, Massachusetts.
