# Supplementary material for: Cranial Remain from Tunisia Provides New Clues for the Origin and Evolution of Sirenia (Mammalia, Afrotheria) in Africa
Source: PLoS One. 2013 Jan 16;8(1):e54307. doi: 10.1371/journal.pone.0054307 (PMC3546994; doi:10.1371/journal.pone.0054307)
Supplement: Information S2 — Ray tooth description. (DOC) [file pone.0054307.s002.doc]

S2. Ray tooth description.

**Torpediniformes Compagno, 1973**

***?Narcine* sp.**

**Description**: This tooth measures 3 mm in width and its root is partially preserved. The crown is laterally elongated and cuspidate, having probably belonged to an antero-lateral file. Labial and lingual ornamentations lack on the totally of the enameloid surface of crown. Only streaks of wear are noticed on oral and labial views (Fig.1D, E). Labial face is slightly convexe from the apex of the short cusp to the labial visor that largely overlaps the root-crown boundary in profile (Fig.1C). The labial visor marks a transversal rounded bulge, visible in basal view (Fig.1D). The transversal crest between lingual and labial face is not sharp but distinct in lingual view (Fig.1A). Lingual face is strait and its basal edge is sinuous in lingual view. The root is high with flattened lobes separated by a broad groove. Other features are unobservable due to the wrong state of preservation, especially the singular orientation of nutritive foramen in Torpediniformes.

**Discussion***:* Cappetta [1] largely figured teeth of all the extant genera of Torpediniformes. The tooth morphology (with the presence a large flattened labial face) reminds that one can preferentially observe in some species of *Narcine* compared to *Torpedo* for instance, even if such distinction must be considered with care considering the wrong state of preservation of the unique tooth. This fossil resembles in some aspect to the material reported by Cappetta as *Narcine* sp. from the late Ypresian of Ouled Abdoun, Morocco [1]. This new occurrence confirms the large Tethysian distribution of Narcinidae (currently limited to tropical western Atlantic and Pacific areas) during the Early Eocene [1].

1. Cappetta H (1988) Les Torpédiniformes (Neoselachii, Batomorphii) des Phosphates du Maroc. Observation sur la denture des genres actuels. *Tertiary Res*. 10: 21-52.


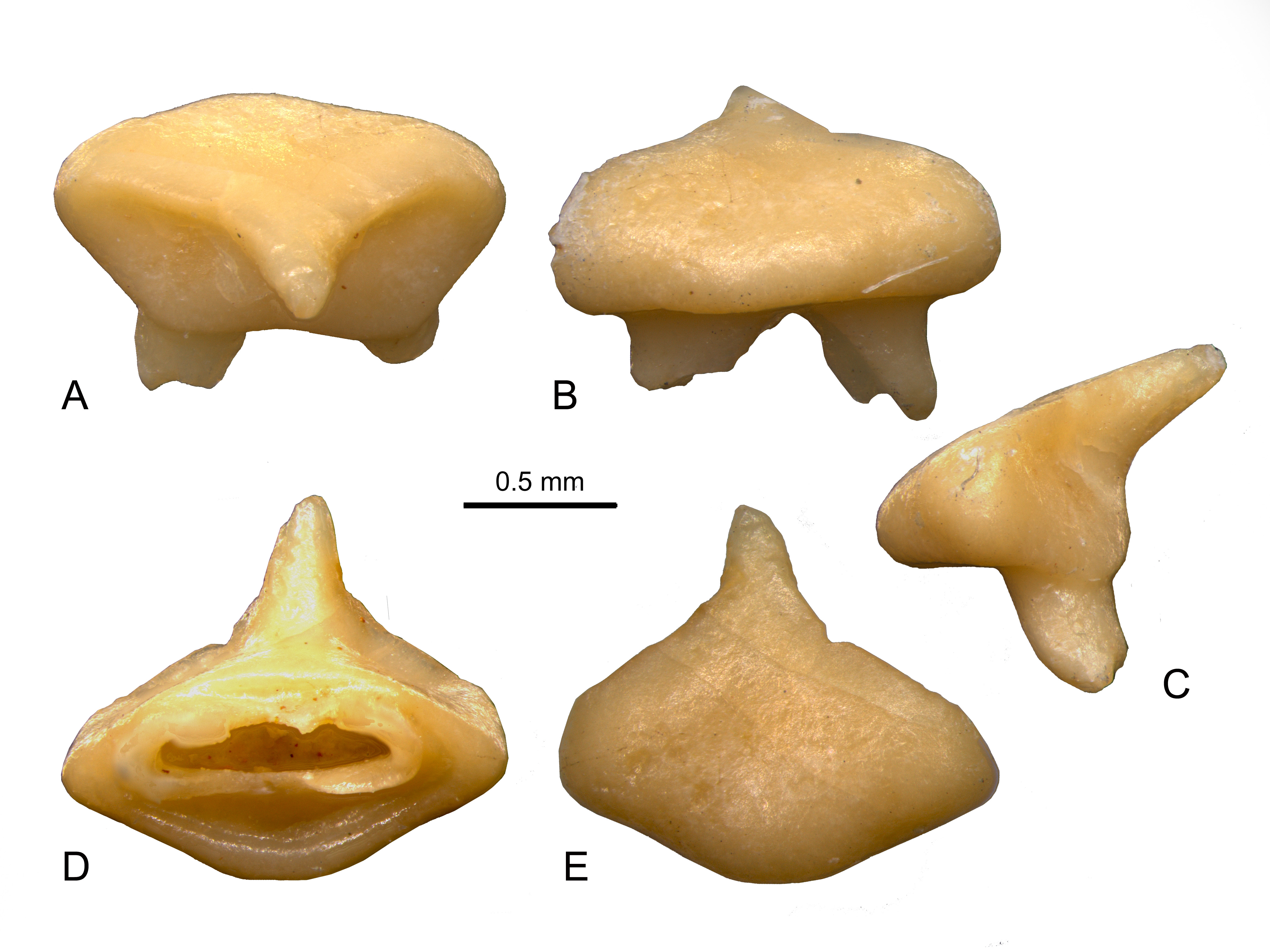


Figure 1. *?Narcine* sp. CB1-561 A. lingual view, B. labial view, C. profile, D. basal view and E. oral view.
