# Supplementary material for: Cranial Remain from Tunisia Provides New Clues for the Origin and Evolution of Sirenia (Mammalia, Afrotheria) in Africa
Source: PLoS One. 2013 Jan 16;8(1):e54307. doi: 10.1371/journal.pone.0054307 (PMC3546994; doi:10.1371/journal.pone.0054307)
Supplement: Information S3 — Measurement protocol. (DOC) [file pone.0054307.s003.doc]

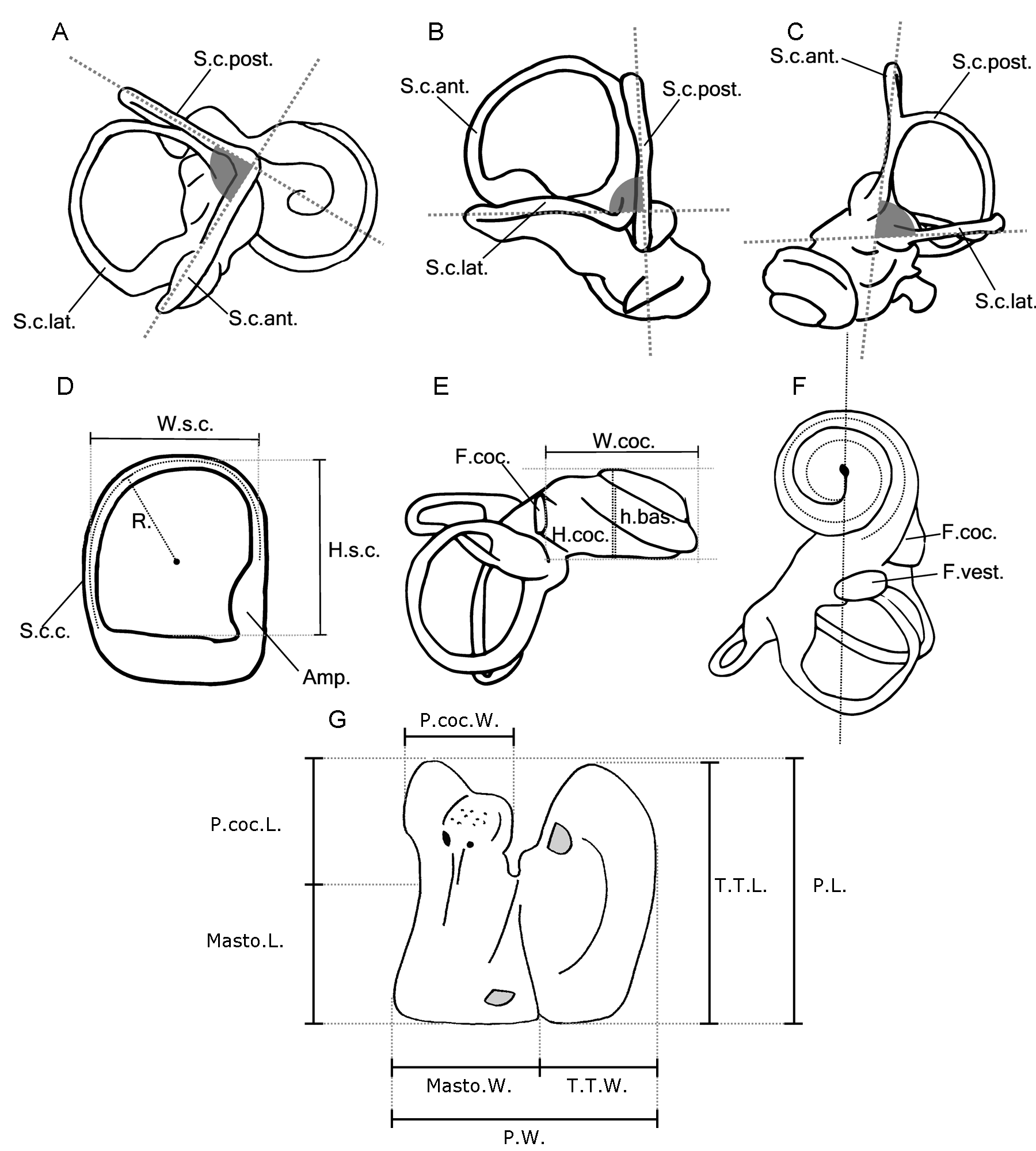


S3. Measurement protocol. A. Angle between the posterior and anterior semicircular canals. B. Angle between the posterior and lateral semicircular canals. C. Angle between the lateral and anterior semicircular canals. D. Measurements of the width, height and radius of semicicular canals. E. Measurements of the height and width of the cochlear canal. The height of the basal turn was measured as the height of the first turn of the cochlear canal at the level of the greatest height of the cochlear canal. F. Coiling of the cochlear canal. The cochlear aspect ratio corresponds to the quotient between the width and the height of the cochlear canal in profile. The arc radius of a canal is half the average of the height and width of the arc. The height of a semicircular canal was measured as the greatest distance from the wall of the bony vestibule to the centre of the lumen of the canal. The width of a semicircular canal was taken perpendicular to the respective heights, and measured from the centres of the lumina of the opposing limbs. Legend. Amp.: Ampulla; F.coc.: *Fenestra cochleae*; F.vest.: *Fenestra vestibuli*; H.coc.: Height of the cochlear canal; h.bas.: Height of the basal turn of the cochlear canal; H.s.c.: Height of the semicircular canal; Masto.L.: Length of the mastoid apophysis; Masto.W.: Width of the mastoid apophysis; P.coc.L.: Length of the pars cochlearis; P.coc.W.: Width of the pars cochlearis; P.L.: petrosal length; P.W.: Petrosal width; R.: Radius of the semicircular canal; S.c.ant.: Anterior semicircular canal; S.c.c.: semicircular canal; S.c.lat.: Lateral semicircular canal; S.c.post.: Posterior semicircular canal; T.T.L.: *tegmen tympani* length; T.T.W.: *tegmen tympani* width; W.coc.: Width of the cochlear canal; W.s.c.: Width of the semicircular canal.
